# Extraction of HCV-RNA from Plasma Samples: Development towards Semiautomation

**DOI:** 10.1155/2015/367801

**Published:** 2015-03-19

**Authors:** Imran Amin, Tania Jabbar, Fawad Niazi, Muhammad Saeed Akhtar

**Affiliations:** ^1^PCR Laboratory, Punjab Institute of Nuclear Medicine (PINUM), Faisalabad 2019, Pakistan; ^2^Department of Clinical Pathology, Punjab Institute of Nuclear Medicine (PINUM), Faisalabad 2019, Pakistan; ^3^Nuclear Medicine Department, Punjab Institute of Nuclear Medicine (PINUM), Faisalabad 2019, Pakistan

## Abstract

A semiautomated extraction protocol of HCV-RNA using Favorgen RNA extraction kit has been developed. The kit provided protocol was modified by replacing manual spin steps with vacuum filtration. The assay performance was evaluated by real-time qPCR based on Taqman technology. Assay linearity was confirmed with the serial dilutions of RTA (Turkey) containing 1 × (10^6^, 10^5^, 10^4^, and 10^3^) IU mL^−1^. Comparison of test results obtained by two extraction methods showed a good correlation (*r* = 0.95, *n* = 30) with detection limit of 10^2^ IU mL^−1^. The semiautomated vacuum filtration based protocol demonstrated high throughput: 35 minutes for the extraction of a batch of 30 samples (150 *µ*L each) with reduced labor, time, waste, and cost. Performance characteristics of semiautomated system make it suitable for use in diagnostic purpose and viral load determinations.

## 1. Introduction

The extraction process is a key component of nucleic acid detection, as it affects both the reliability and the reproducibility of target amplification. The manual extraction method (spin based) using Favorgen extraction RNA kit is time consuming and requires meticulous technical skills to achieve reproducible results [1, 2]. Therefore, demand for automated system has grown in recent years. Automated nucleic acid extraction process is potentially beneficial to reduce working time, labor cost, and risk of contamination and at the same time increases the worker safety and laboratory efficiency [3]. However, fully automated extraction system is very expensive and raises the cost of diagnosis. Therefore, developments towards semiautomation (vacuum filtration based protocol) not only reduce labor, plastic waste, and price (100 Pak Rs. or 1 US dollar) but also speed up the extraction process.

Although the introduction of real-time PCR has led to considerable progress in automating the amplification and detection steps, still nucleic acid isolation remains very labor-intensive when performed manually. Thus, the objective of the present study was to evaluate performance of vacuum manifold system and spin based protocols for extraction of HCV-RNA using Favorgen kit high efficiency silica based spin column as reported in [4] followed by RT-qPCR. The sensitivity of two methods was compared using samples of different viral load.

## 2. Materials and Methods

**Figure 1:**
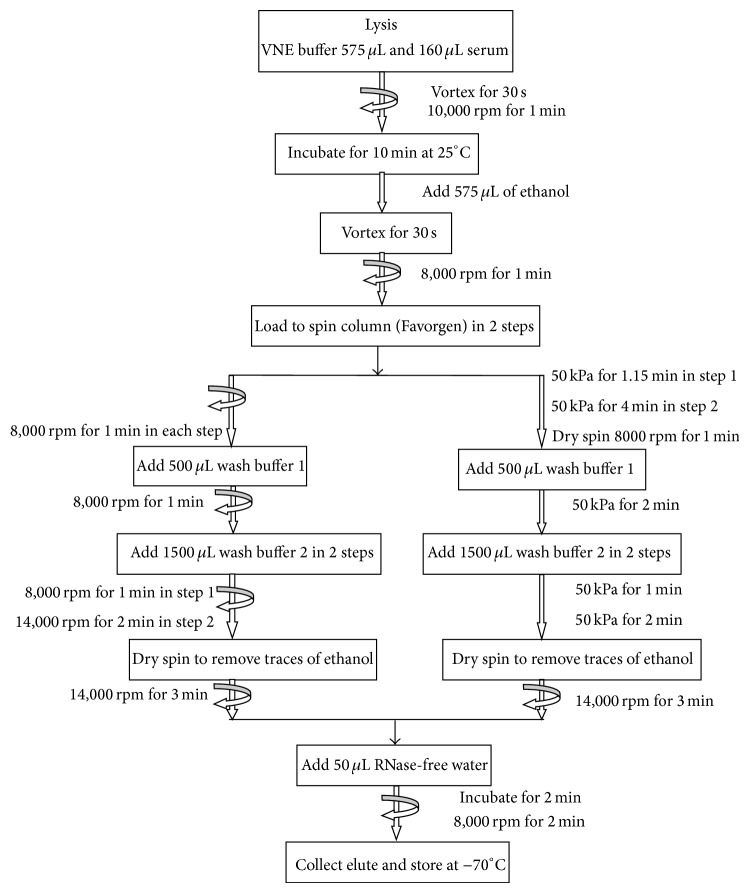
Spin and vacuum filtration based protocol for HCV-RNA extraction using Favorgen kit.

In the present study, 30 EDTA anticoagulated plasma samples submitted to the Punjab Institute of Nuclear Medicine for quantitative analysis of HCV were processed. HCV-RNA had been extracted by use of the Favorgen kit according to the manufacturer's instructions [5] as shown in Figure 1. In 2 mL microcentrifuge tubes, 150 *μ*L plasma samples were mixed with 575 *μ*L of lysis buffer and appropriate amounts of internal controls. After incubation for 12 min, 575 *μ*L of ethanol was added to the tube for precipitation. The samples were vortexed (vortex mixer Labnet) and centrifuged (Eppendorf centrifuge 5424). Subsequently, the working solution was loaded into spin column in two steps and was separated by centrifugation at 8000 rpm (RCF) for 1 min. To remove cell debris, RNA attached to silica membrane was washed with buffers 1 and 2. After complete removal of the final washing buffer by centrifugation at 14000 rpm for 3 min, RNA was eluted with RNase-free water, and the eluate was stored at −70°C. At least 30 samples could be processed in 60 min.

### 2.1. Semiautomated Extraction Protocol

To permit a higher throughput, the manual protocol described above was implemented on vacuum filtration assembly (Welch-Ilmvac 2522) under conditions as shown in Figure 1.

### 2.2. RT-qPCR

HCV-RNA was quantified using real-time Taqman based AmpliSens HCV-FRT kits [6]. A 15 *μ*L master mix containing RT-G-mix-2, RT-PCR-mix-1, RT-PCR-mix-2, polymerase (TaqF), and TM-Revertase (MMIv) was added to 10 *μ*L of each eluted nucleic acid.

RT-qPCR was completed on Rotor-Gene Q 6000 (5 Plex-HRM) using the following cycling parameters: an initial cDNA synthesis by holding at 50 and 95°C for 15 min each, followed by 45 cycles of denaturation (95°C for 2 s), annealing (60°C for 5 s), and extension (72°C for 15 s). Mathematical analysis and graphical representations were performed using Rotor-Gene Q software.

## 3. Results and Discussions

Almost 30 clinical samples were subjected to RNA extraction in parallel by the spin and vacuum filtration based protocol using Favorgen kit. The experimental conditions of vacuum filtration were optimized by repeating experiments using positive control. RT-qPCR results by two extraction methods are given in Table 1. All the samples were run in duplicate and mean value was used for data analysis. Assay linearity was confirmed with the serial dilutions of RTA (Turkey) containing 1 × (10^6^, 10^5^, 10^4^, and 10^3^) IU mL^−1^. Viral load HCV-RNA was expressed in IU mL^−1^. Although the difference of viral load was not very dramatic but rather high, yield for the sera with amplified product was obtained by vacuum filtration based protocol with coefficient of variation of 6.4% [7]. One of the reasons that might be possible for high HCV-RNA yield is strong retention capacity of HCV-RNA on silica based membrane under employed vacuum pressure [8]. Constant amplification efficiency made the comparison reliable between two assays as demonstrated by threshold cycle of internal control CT IC.

**Figure 2:**
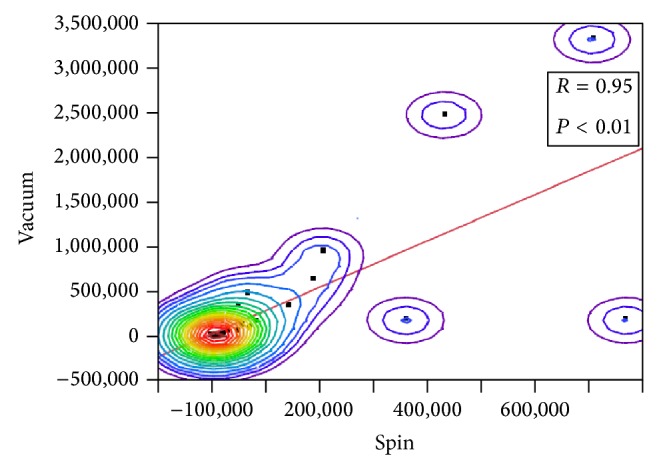
Bivariate fit between two assays.

A bivariate normal distribution fit between two assays gave diagonally distributed density ellipsoid with correlation of 95% between vacuum filtration and spin based protocols, indicating that both methods were efficient in the removal of inhibitory substance; see Figure 2.

Nevertheless, the modification of protocol from spin to vacuum filtration allowed extraction to be completed within 35 minutes. In addition, costs for consumables in semiautomated vacuum filtration based extraction reduced from $1 to 0.5 with reduced labor. At the same time, this protocol is more environmentally friendly due to reduced incineration of infected collection tubes. Thus, semiautomation is a more forward looking approach for nucleic acid purification together with real-time qPCR.

## 4. Conclusion 

The performance of semiautomated vacuum filtration based extraction method was shown to permit a quick extraction process and accurate results for a quantitative assay of HCV-RNA. The method might be an alternative to an expensive full automation station for developing countries which is easy to perform and efficient. In short, costly instruments are not required to prevent contamination and to enhance the safety of worker.

## Figures and Tables

**Table 1:** RT-qPCR results using spin and vacuum filtration based protocols for HCV-RNA extraction.

Sample ID	CT Internal control	CT	CT	IU mL^−1^	IU mL^−1^	Cv %
		Vacuum filtration	Spin	Vacuum filtration	Spin	
1	25.99	15.6 ± 0.3	18.4 ± 0.4	481,419	67,458	0.9
2	26.48	16.0 ± 0.3	17.0 ± 0.3	360,304	177,932	0.6
3	26.57	22.6 ± 0.7	24.7 ± 0.5	3,602	847	0.01
4	26.8	12.8 ± 0.2	15.1 ± 0.3	3,299,175	704,535	6.4
5	27.5	18.6 ± 0.4	19.6 ± 0.4	60,629	28,823	0.1
6	26.34	13.3 ± 0.2	15.7 ± 0.3	2,461,014	430,986	4.7
7	27.14	16.1 ± 0.3	18.8 ± 0.4	348,791	51,043	0.7
8	26.86	17.6 ± 0.3	18.6 ± 0.4	112,812	56,348	0.2
9	27.84	14.6 ± 0.2	16.8 ± 0.3	946,830	206,548	1.8
10	26.54	17.2 ± 0.3	31.8 ± 1.2	160,482	54,132	0.3
11	26.29	19.2 ± 0.4	20.4 ± 0.6	38,131	16,544	0.1
12	24.9	21.4 ± 0.6	22.18 ± 0.7	9,683	5,736	0.0
13	24.7	15.07 ± 0.3	17.2 ± 0.3	767,124	179,381	0.2
14	26.28	18.9 ± 0.4	19.8 ± 0.4	52,693	29,976	0.02
15	26.77	22.2 ± 0.7	22.6 ± 0.7	5,810	4,340	0.0
16	26.2	20.4 ± 0.6	21.2 ± 0.6	19,312	11,584	0.01
17	25.4	19.8 ± 0.4	20.0 ± 0.4	29,849	25,136	0.01
18	26.8	21.6 ± 0.6	21.8 ± 0.7	8,328	7,507	0.00
19	26.1	15.3 ± 0.3	17.1 ± 0.3	642,090	187,782	0.2
20	26.0	21.4 ± 0.6	23.4 ± 0.7	9,533	2,555	0.0
21	26.1	18.8 ± 0.4	19.0 ± 0.4	55,731	50,456	0.02
22	26.1	17.2 ± 0.3	18.3 ± 0.4	171,609	83,106	0.06
23	25.7	19.3 ± 0.4	20.1 ± 0.4	46,845	24,354	0.02
24	28.4	21.5 ± 0.6	22.5 ± 0.7	9,201	4,760	0.0
25	26.1	22.1 ± 0.6	24.3 ± 0.7	5,980	1,307	0.0
26	26.8	20.3 ± 0.6	20.7 ± 0.6	20,985	16,260	0.01
27	31.5	26.0 ± 0.9	26.9 ± 0.8	280	150	0.01
28	18.5	18.1 ± 0.3	18.5 ± 0.4	95,495	70,695	0.04
29	31.2	16.2 ± 0.3	17.5 ± 0.3	352,282	145,151	0.1
30	27.5	19.44 ± 0.4	20.5 ± 0.6	37,850	18,256	0.01
