# Corrigendum to “Extraction of HCV-RNA from Plasma Samples: Development towards Semiautomation”

**DOI:** 10.1155/2016/3278363

**Published:** 2016-11-29

**Authors:** Imran Amin, Tania Jabbar, Fawad Ali, Muhammad Saeed Akhtar

**Affiliations:** ^1^PCR Laboratory, Punjab Institute of Nuclear Medicine (PINUM), Faisalabad 2019, Pakistan; ^2^Department of Clinical Pathology, Punjab Institute of Nuclear Medicine (PINUM), Faisalabad 2019, Pakistan; ^3^Nuclear Medicine Department, Punjab Institute of Nuclear Medicine (PINUM), Faisalabad 2019, Pakistan

In the article titled “Extraction of HCV-RNA from Plasma Samples: Development towards Semiautomation” [1], the name of the third author was given incorrectly as Fawad Niazi. The author's name should have been written as Fawad Ali. The revised author list is shown above.
